# A novel case of tracheal deviation secondary to percutaneous tracheostomy

**DOI:** 10.1186/s44215-022-00023-0

**Published:** 2023-03-20

**Authors:** Lily Wang, Justin Lu, Michael A. Ko

**Affiliations:** 1 Department of Otolaryngology – Head & Neck Surgery, University of Toronto, Toronto, Ontario Canada; 2 Temerty Faculty of Medicine, University of Toronto, Medical Sciences Building, 1 King’s College Cir, Toronto, ON M5S 1A8 Canada; 3 Department of Surgery, St. Joseph’s Health Centre, University of Toronto, Toronto, Ontario Canada

**Keywords:** Tracheostomy, Subglottic stenosis, COVID-19

## Abstract

**Background:**

This is a novel case of iatrogenic airway stenosis and tracheal deviation in a patient with previous percutaneous dilational tracheostomy.

**Case presentation:**

This 65-year-old male presented with a short segment of combined flap-valve stenosis and tracheomalacia, with right tracheal deviation distal to the stenosis. Clinical staging of the stenosis corresponded to a Cotton-Meyer grade III and McCaffrey stage III. Tracheal resection and primary reconstruction was performed by thoracic surgery and otolaryngology.

**Conclusion:**

No cases have reported both stenosis and tracheal deviation as a result of iatrogenic intervention. The nature of this injury highlights a need for rigour in approaching tracheostomy.

## Background

COVID-19 has resulted in a substantial increase in the number of tracheostomies performed worldwide [1]. This is commonly accomplished through percutaneous dilational tracheostomy (PDT) at the patient’s bedside for efficiency and cost-effectiveness [2, 3].

Here, we present a novel case of a PDT complication resulting in iatrogenic airway stenosis and tracheal deviation. To our knowledge, this is the first case reported worldwide.

## Case

This 65-year-old man was referred to the thoracic surgery clinic for post-tracheostomy subglottic stenosis (SGS). He had severe COVID pneumonia in the year prior and was ventilated for more than 75 days (intubated 15 days, then tracheostomy). His past medical history was significant for hypertension, type 2 diabetes, and dyslipidaemia.

**Fig. 1:**
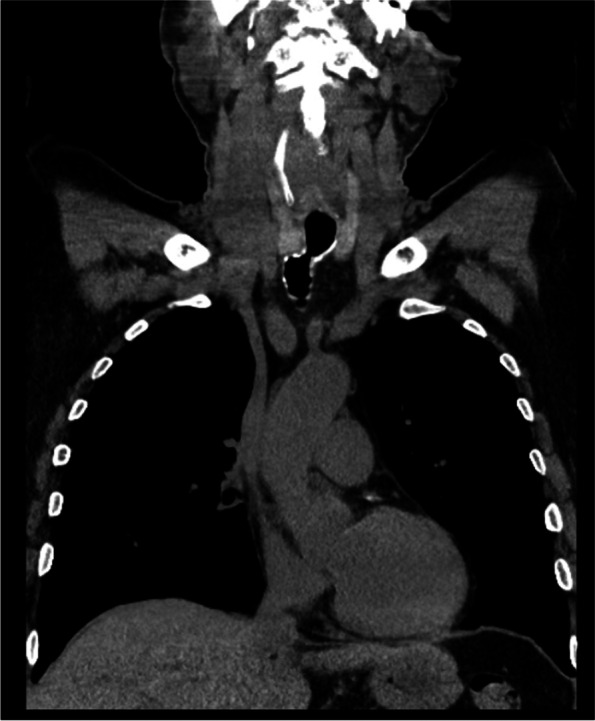
Coronal CT scan. The stenotic segment measured 1.8 cm in length, with a minimal medial-lateral diameter of 0.5 cm and minimal AP diameter of 1 cm measured on the coronal and sagittal inspiratory images

At initial presentation, his main complaint was orthopnoea. He had audible laboured breathing at baseline. The patient recalls the tracheostomy being off centre to the left and experienced a tugging sensation around the insertion site with rotation of his head contralaterally. His CT scan (Fig. 1) demonstrated focal subglottic narrowing and severe tracheal deviation with possible evidence on a previous CT scan. The trachea was deviated towards the right at the level of the stenotic segment, which measured 1.8 cm in length.

**Fig. 2:**
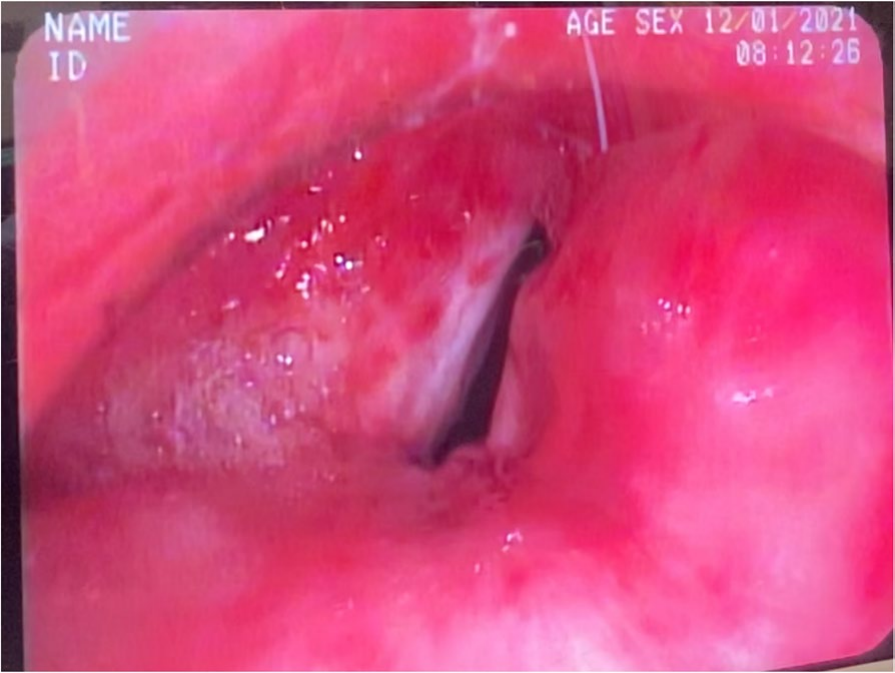
Preoperative bronchoscopic imaging findings

On flexible and rigid bronchoscopy, the glottis was clear, and the vocal cords were mobile bilaterally (Fig. 2). At the proximal trachea, there was a short segment of combined flap-valve stenosis and tracheomalacia, with right tracheal deviation distal to the stenosis. Clinical staging of the stenosis corresponded to a Cotton-Meyer grade III and McCaffrey stage III [4, 5]. Intubation with a 7.5 rigid bronchoscope past the stenosis revealed normal distal airways. The stenosed area was not amenable to dilation. Intraoperative consultation with otolaryngology concluded tracheal resection would be required to treat the stenosis.

**Fig. 3:**
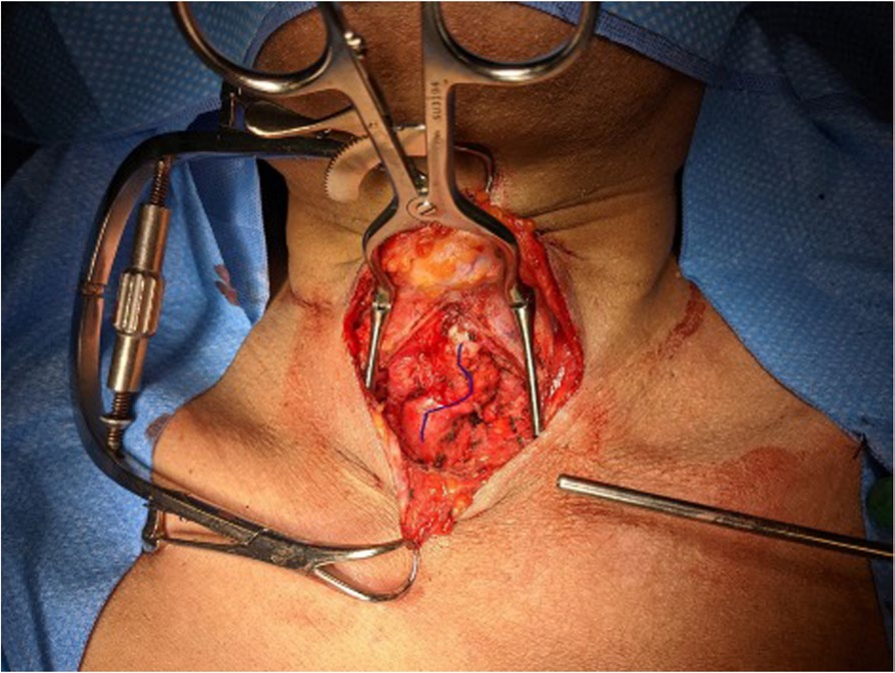
Intraoperative image. Tracheal deviation marked in blue

**Fig. 4:**
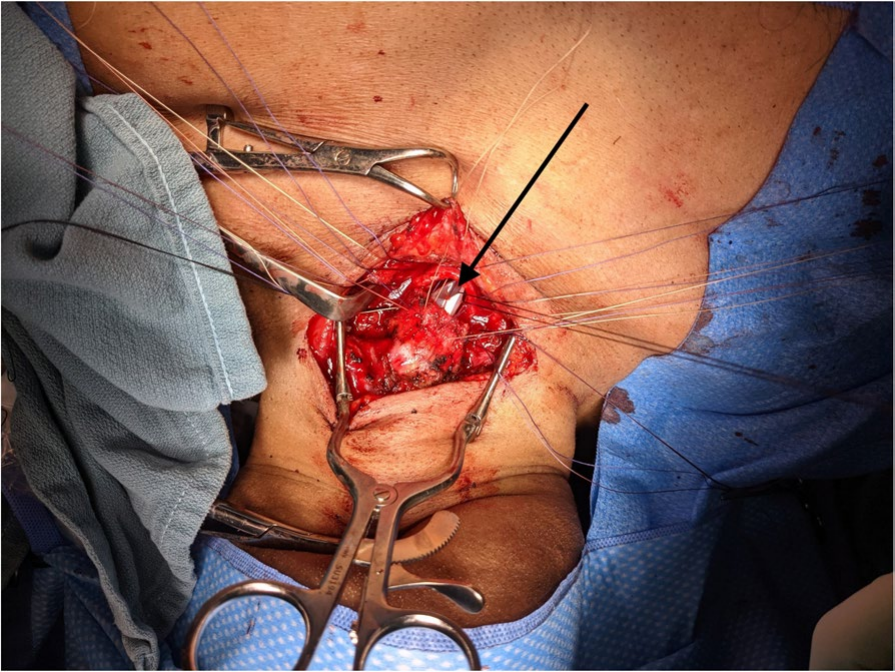
Intraoperative image. Area of original tracheostomy indicated by arrow

Tracheal resection and primary reconstruction was jointly performed by thoracic surgery and otolaryngology 2 weeks following bronchoscopy (Fig. 3). The procedure was performed by dividing the thyroid isthmus and laying open the anterior tracheal wall. Sharp and blunt dissection was performed using Metzenbaum scissors and bipolar cautery, staying close the anterior wall, and limiting the disruption of the lateral vascular supply. Both recurrent laryngeal nerves were identified and preserved. Due to the length of the procedure, a hyoid release was performed as well as mediastinal tracheal mobilisation. The trachea was divided sharply under direct bronchoscopic visualisation with a #15 blade, and cross field sterile ventilation was set up with an armoured endotracheal tube (Fig. 4). Stay sutures of 2-0 silk were used to assess tension on the anastomosis. The anastomosis was performed with a running 3-0 PDS suture for the membranous trachea and interrupted 3-0 Vicryls for the anterior wall. Following extubation in the OR, he had no audible breathing or stridor, and his voice was clear. Unfortunately, the next day, he had episodes of retching and hypoxia and increased neck oedema. Bronchoscopy demonstrated supraglottic and epiglottic oedema and erythema. He was intubated and underwent CT that showed extensive soft tissue inflammation deep within the neck bilaterally and surrounding the trachea. He was empirically started on antibiotics for possible aspiration and IV steroids for post-surgical oedema.

On post-operative day 7, repeat fiberoptic nasopharyngoscopy at bedside showed improvement in his supraglottic oedema, and he was successfully extubated.

This patient was seen postoperatively at 8 months in thoracic surgery clinic and reported a complete resolution of their symptoms and significantly improved quality of life.

## Discussion and conclusions

There are no previous instances of iatrogenic tracheal deviation secondary to prolonged tracheostomy reported in the literature [6–11]. Review of this patient suggests sidewall entry into the trachea during PDT created an area of focal stenosis which caused distal trachea deviation. Prolonged cuff pressure from the tracheostomy tube at such an angle contributed to trachea necrosis, cartilage exposure, circumferential scarring, and ultimately stenosis. Following decannulation, the patient had collapse of his tracheal cartilage around the area of stenosis due to tracheomalacia, causing his stridorous symptoms.

The incidence of iatrogenic airway stenosis in the literature is approximately 10–31% of patients [12]. Cases of concurrent airway stenosis and tracheal deviation include compression of the airways from nearby cancer or thyroid goitre. One case describes a patient with a 90° tracheal deviation at the level of severe spinal kyphoscoliosis [9, 11, 13]. Tracheal stenosis was distal to the site of deviation in these cases and did not cause significant respiratory symptoms, and the stenosis was ultimately reversed with treatment of underlying cause. In contrast, our patient’s stenosis was an iatrogenic complication from PDT and ultimately required surgical resection.

This case history outlines the need for increased care surrounding PDT procedures as it may result in significant long-term consequences for patients. The presence of patient comorbidities including cardiovascular disease, tube characteristics (size, cuff pressures, duration of insertion), and perioperative management (sedation or local infections) all modify risk for developing airway stenosis [1]. In this case, tracheostomy insertion angle also significantly contributed to the development of his respiratory distress. Surgeons should take great care to adhere to a midline approach during PDT given the poor visualisation of the patient’s anatomy in comparison to open tracheostomy. To prevent such complications in the future, checklists, timeouts, or quality improvement initiative programmes may be considered and have been implemented in other bedside procedures with effect [14].

Cases of tracheal stenosis combined with tracheal distortion provide a unique management challenge unlike cases of simple stenosis. Routine interventions like tracheal dilation and tracheobronchial airway stents are less effective as they are not malleable to the contours of tracheal distortion as is the case in the patient presented in this report. Moreover, patients with tracheal distortion have an additional cause of airway narrowing on top of the stenosis that is not correctable with dilation or stent alone.

This case describes a patient requiring surgical resection for iatrogenic airway stenosis resulting in distal tracheal deviation. The complex nature of this injury highlights a need for rigour in approaching tracheostomy. Further investigations are also required to assess the relationship between tracheostomy entry angle and risk of tracheal deviation.

## Data Availability

All data generated in this study are included in the final published article.

## Abbreviations

CT: Computerized tomography
COVID-19: Coronavirus disease 2019
REB: Research Ethics Board
IV: Intravenous
PDT: Percutaneous dilational tracheostomy
SGS: Subglottic stenosis
